# Supplementary material for: Integration of multiple biological contexts reveals principles of synthetic lethality that affect reproducibility
Source: Nat Commun. 2020 May 12;11:2375. doi: 10.1038/s41467-020-16078-y (PMC7217969; doi:10.1038/s41467-020-16078-y)
Supplement: Supplementary file 1 — Supplementary Information [file 41467_2020_16078_MOESM1_ESM.pdf]

Supplementary Information for

# Integration of multiple biological contexts reveals principles of synthetic lethality that affect reproducibility

Angel A. Ku[1], Hsien-Ming Hu[1], Xin Zhao[1], Khyati N. Shah[1], Sameera Kongara[1], Di Wu[2], Frank McCormick[2], Allan Balmain[2], Sourav Bandyopadhyay[1,2]

Correspondence to: Sourav.bandyopadhyay@ucsf.edu

## SUPPLEMENTARY FIGURES

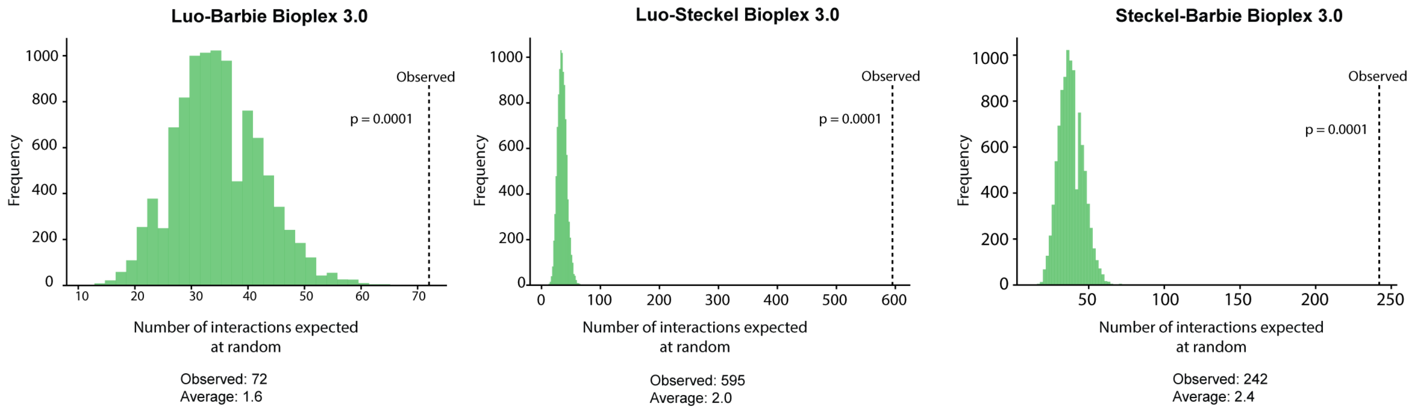


**Supplementary Figure 1: Comparison of hits from KRAS SL studies using the Huttlin et al. 2020 BioPlex PPI network.** Comparison of the number of interactions observed using the Huttlin et al. protein-protein interaction (PPI) network spanning between hits reported in the two indicated studies versus the number of similar interactions observed between random genes. Histogram represents results from 10,000 simulations conducted by randomly picking 250 genes that were tested in each respective study and the p-value represents the fraction of simulations where the same or more interactions than the actual observed number were obtained (see Methods).

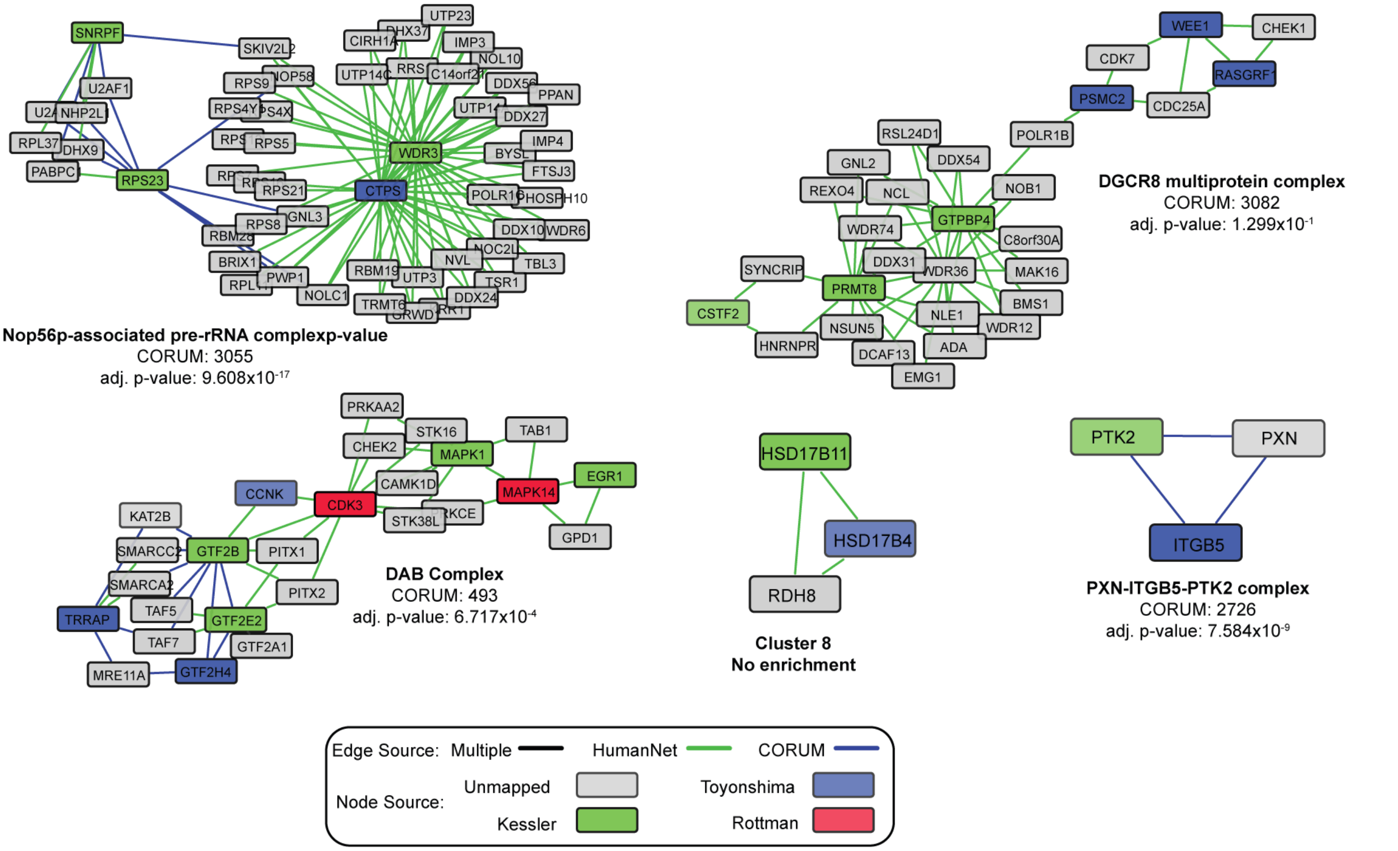


**Supplementary Figure 2: MYC synthetic lethal networks.** Top 250 MYC synthetic lethal genes were taken from large scale RNAi screens from Kessler et al., Rottman et al. and Toyonshima et al. and combined using the human protein-protein interaction network to identify subnetworks identified by multiple studies and then analyzed for enrichment using gProfiler against known protein complexes in CORUM and HumanNet. P-values are based on hypergeometric overlap with CORUM complexes after adjusting for multiple testing.

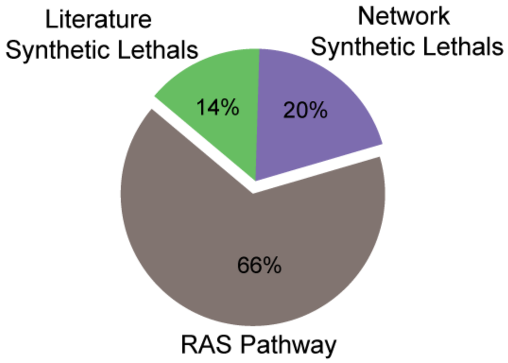


**Supplementary Figure 3: Distribution of 196 genes tested in esiRNA screen.** Individual genes are listed in Supplementary Data 4.

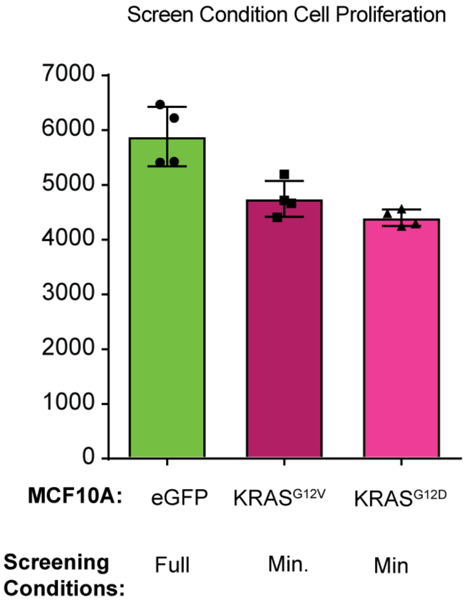


**Supplementary Figure 4: Growth rate between eGFP cells in full media and KRAS cells in minimal media.** 1000 Cells were plated in the indicated conditions after 72 hours cells were fixed and nuclei counted. n=4 biologically independent samples. Data are mean and error bars are s.d.

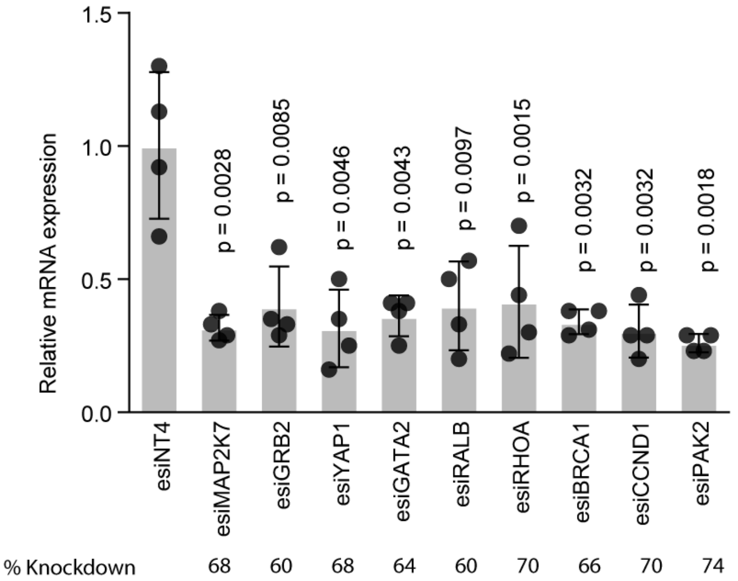


**Supplementary Figure 5: Knockdown efficiency esiRNAs measured by RT-PCR.** Genes were knocked down in MCF10A G12V cells for 48 hours and transcript levels measured by RT-PCR. Percent knockdown compared to non-targeting (NT) is shown. n=4 biologically independent samples. P-values are based on a two-tailed Students t-test. Data are mean and error bars are s.d.

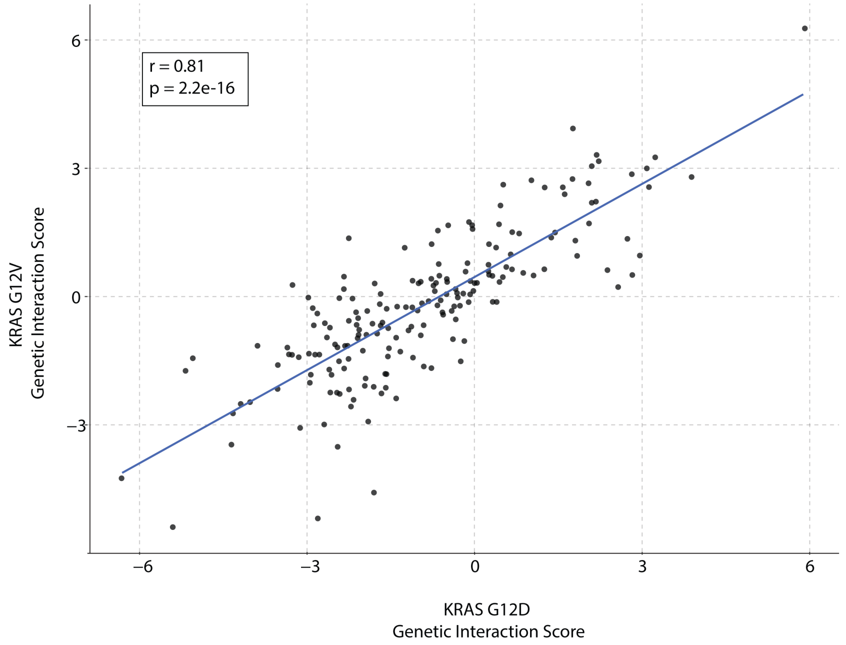


**Supplementary Figure 6: Comparison of KRAS G12V and G12D screens.** Isogenic KRAS G12V and KRAS G12D expressing MCF10A lines were screened using the same esiRNA library and scores for genes compared. P-value of Pearson's correlation (r) is shown.

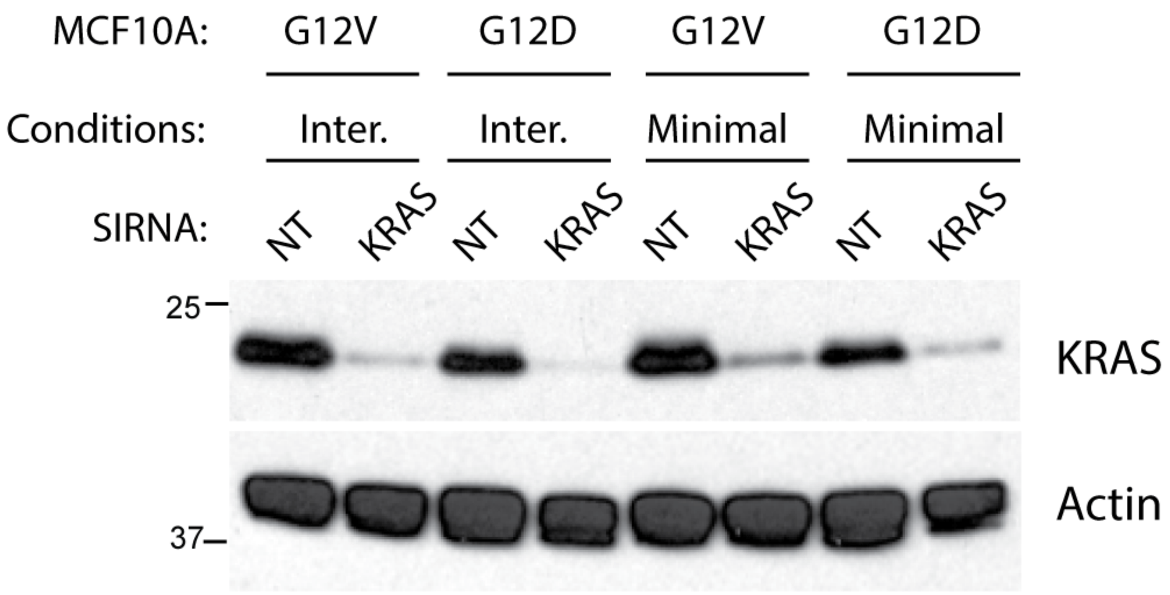


**Supplementary Figure 7: Knockdown efficiency is not impacted by changes in media conditions.** The indicated MCF10A cell line expressing KRAS G12D or G12V mutation were grown in the indicated condition and transfected with non-targeting (NT) or KRAS esiRNA for 48 hours and the resulting cells lysed and subjected to immunoblot. Experiment was repeated twice with similar results.

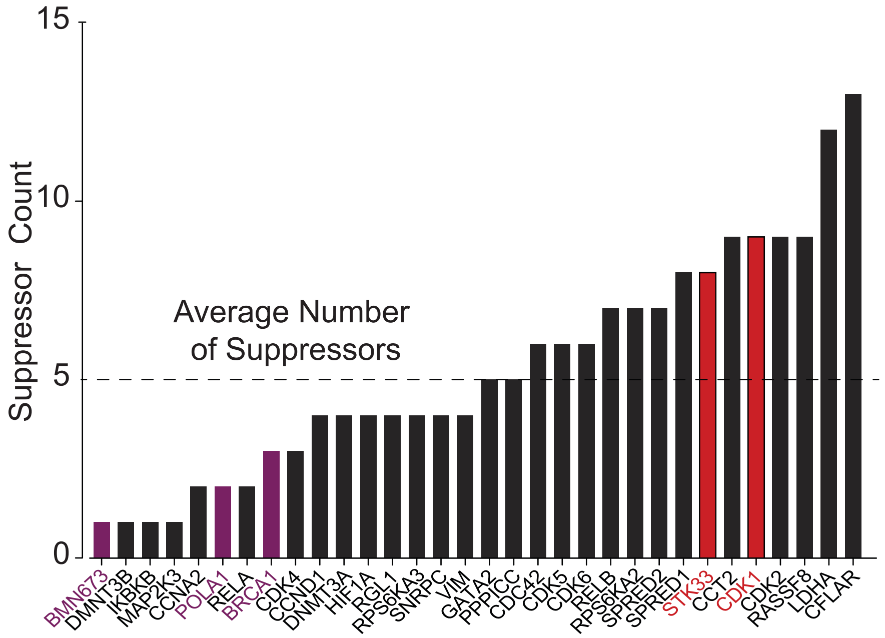


**Supplementary Figure 8: Suppressors of synthetic lethal genes.** Histogram of the number of suppressor genes identified for KRAS synthetic lethal genes and talazoparib. Suppressors defined as genes with a *Z*-score > 2. Purple gene highlight network genes from the cell cycle/replication cluster, red genes highlight two published KRAS synthetic lethal genes with suppressor counts greater than average.

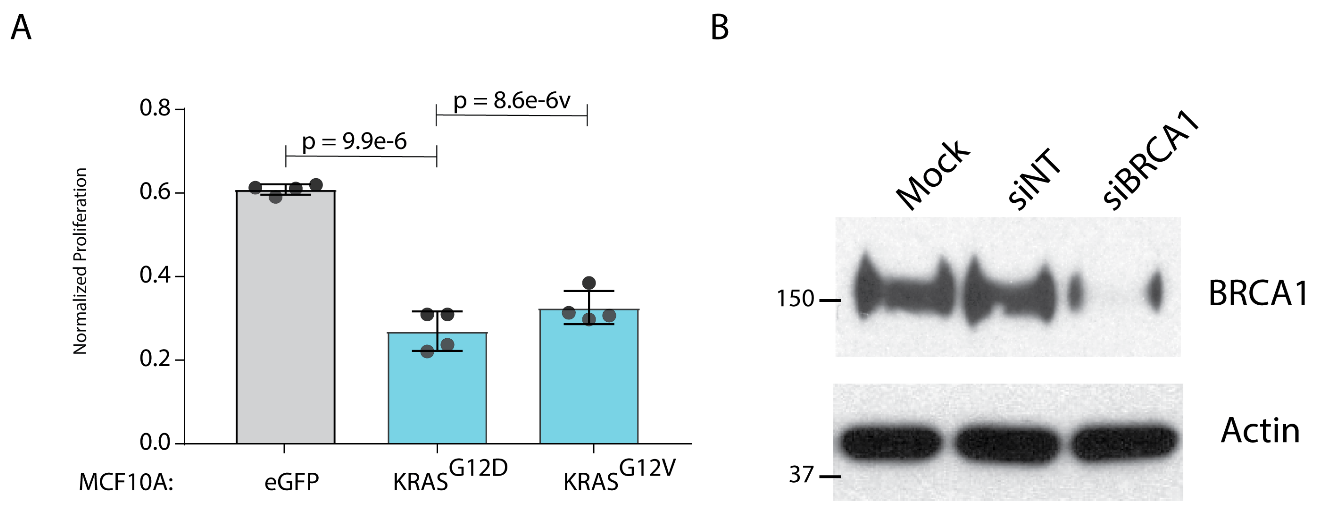


**Supplementary Figure 9: siRNA mediated validation of BRCA1 dependency in mutant KRAS cells.** (A) The indicated MCF10A isogenic cell lines were transfected with BRCA1 siRNA and proliferation measured after 72 hours by nuclei count. Data are proliferation relative to control non-targeting knockdown. n=4 biological independent samples. (B) Western blot of total BRCA1 after knockdown for 48 hours in MCF10A KRAS G12V cells. Experiment was repeated twice with similar results. P-values based on two-sided t-test. Data are mean and error bars s.d..

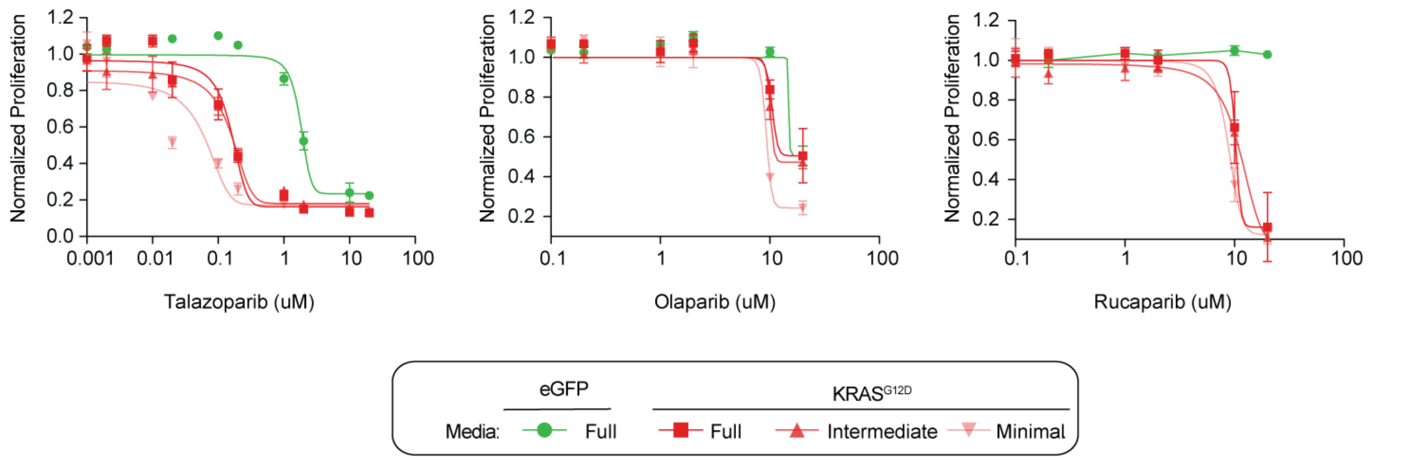


**Supplementary Figure 10: PARP inhibitor sensitivity is independent of media conditions.** Relative proliferation of control eGFP and KRAS G12D MCF10A lines after treatment with PARP inhibitors talazoparib, rucaparib or olaparib for 96 hours in full, intermediate or minimal media conditions. n=4 independent biological samples. Data are mean and error bars s.d.

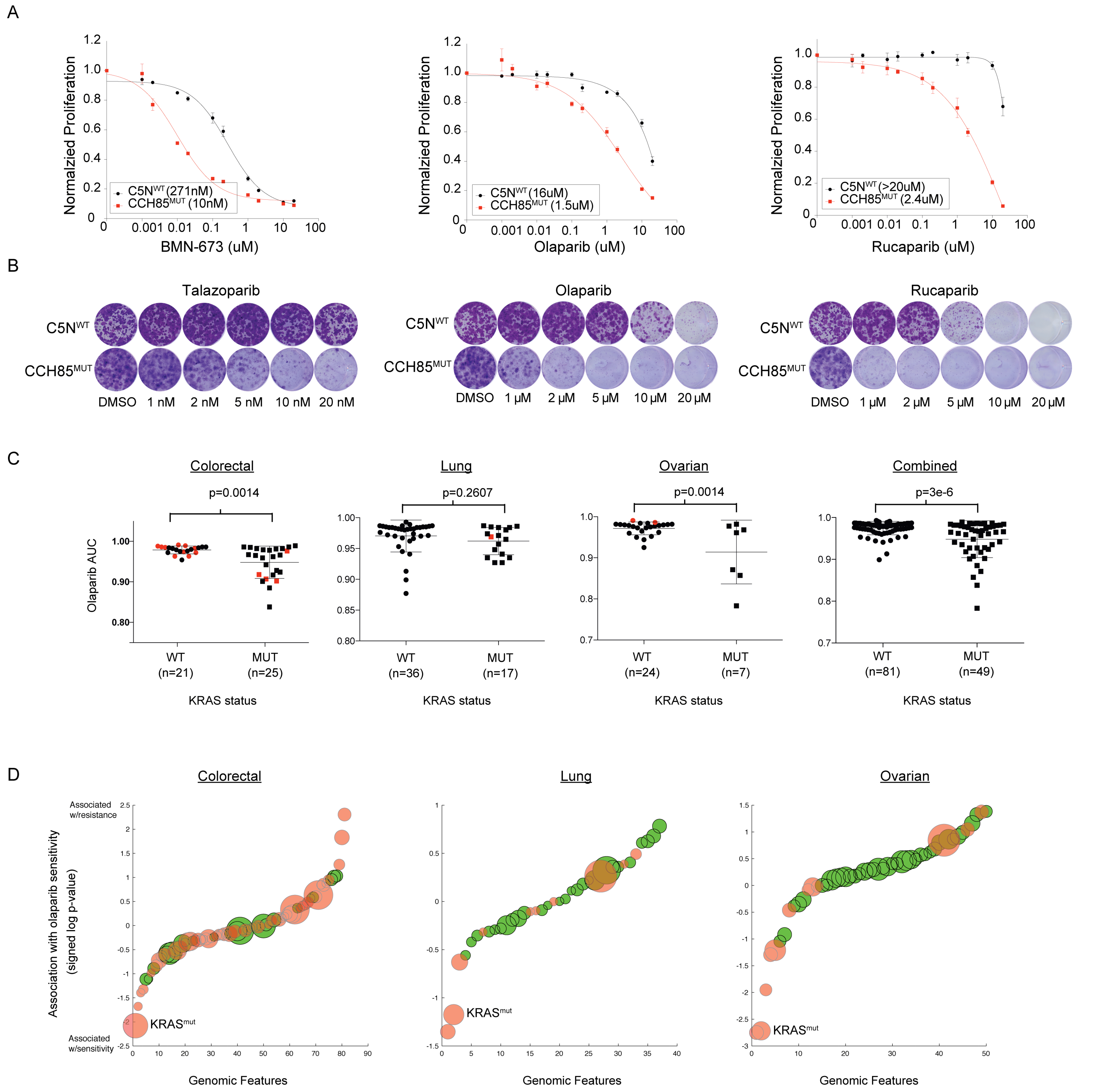


**Supplementary Figure 11: Olaparib drug response analysis in cancer cell lines collections.** (A) Relative proliferation of RAS wild-type C5N skin keratinocyte cells or carcinogen induced CCH85 HRAS-mutant skin carcinoma cells in the presence of PARP inhibitors for 72 hours. IC50s are indicated. n=4 biologically independent samples. (B) Long-term clonogenic growth of the same cell lines treated with PARP inhibitors for 9 days. (C) Cell lines from the three different tumor types that harbor >5 KRAS

mutant and >5 wild-type cell lines were analyzed with respect to olaparib sensitivity in the genomics of drug sensitivity database (GDSC). Responses were compared based on drug area under the dose response curve (AUC) analysis with lower values indicating more drug sensitivity. Shown are responses for each tumor type individually as well as all three combined. The number of biologically independent cell lines in each category is indicated in parenthesis. P-values based on a two-sided t-test. Red dot indicates cell lines with known BRCA1/2 mutations. (D) Associations of genomic features with olaparib sensitivity downloaded from the GDSC database. P-values of association were converted into a signed score by taking the log of the p-value and adding a sign to indicate association with sensitivity (negative values) and association with resistance (positive values). Mutation based features in red and copy-number based features in green. Data are mean and error bars s.d. Not significant, n.s.

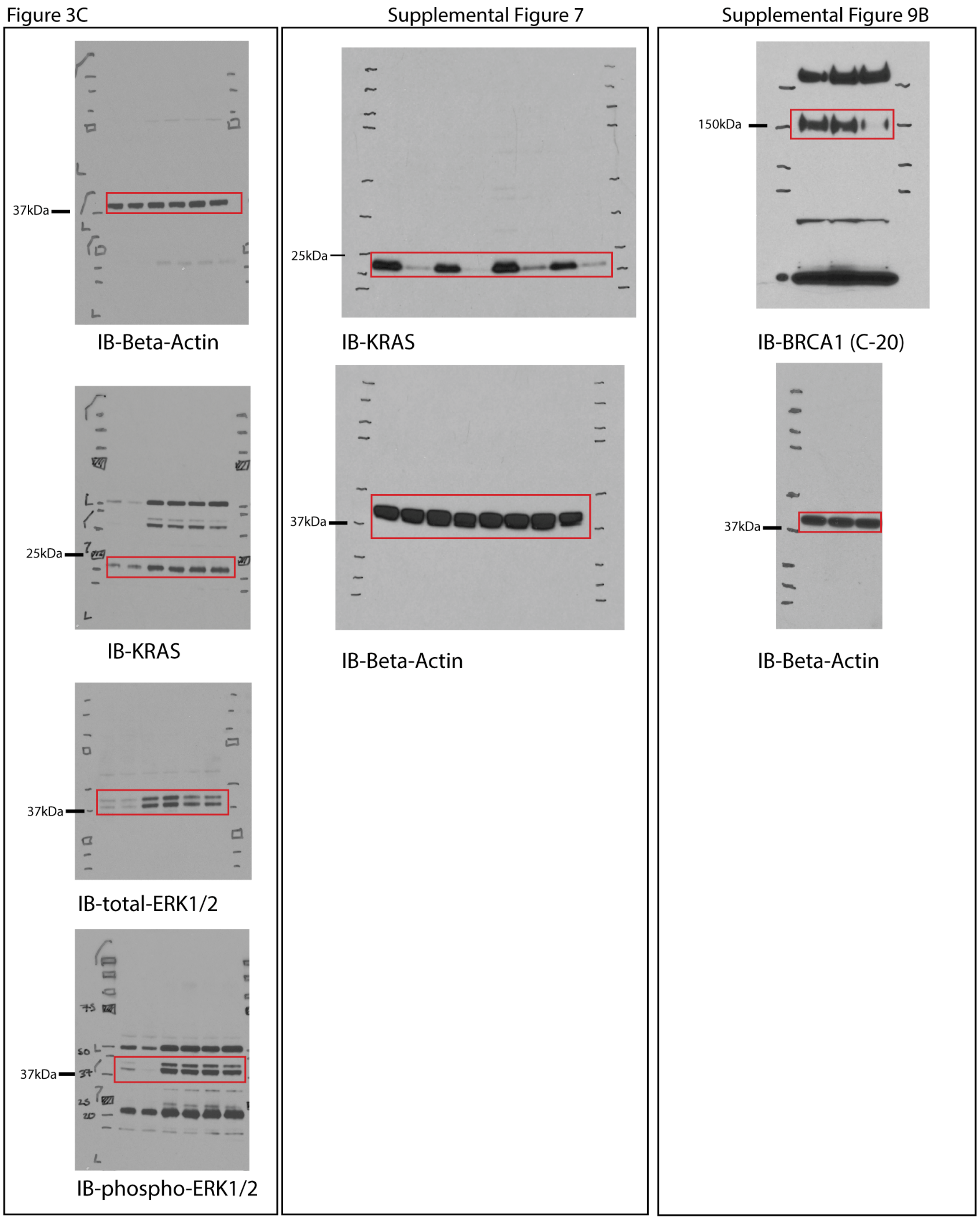


**Supplementary Figure 12: Uncropped immunoblots organized by figure.**
