# Supplementary material for: Integration of multiple biological contexts reveals principles of synthetic lethality that affect reproducibility
Source: Nat Commun. 2020 May 12;11:2375. doi: 10.1038/s41467-020-16078-y (PMC7217969; doi:10.1038/s41467-020-16078-y)
Supplement: Supplementary file 3 — Description of Additional Supplementary Files [file 41467_2020_16078_MOESM3_ESM.pdf]

Description of Additional Supplementary Files

File Name: Supplementary Data 1
Description: Gene lists used for network analysis.

File Name: Supplementary Data 2
Description: List of genes in KRAS synthetic lethal networks, their source and association with protein complexes or pathways and list of KRAS synthetic lethals and their source.

File Name: Supplementary Data 3
Description: Cytoscape session of network results from integration of Barbie, Steckel and Luo KRAS SL studies. Can be opened using Cytoscape (cytoscape.org).

File Name: Supplementary Data 4
Description: esiRNA library genes and annotations.

File Name: Supplementary Data 5
Description: MCODE network results of KRAS SL studies & DepMap CRISPR screens using CORUM complexes.

File Name: Supplementary Data 6
Description: Cytoscape session of network results from integration of 6 siRNA and 2 CRISPR SL studies from the Dependency Map. Can be opened using Cytoscape (cytoscape.org).

File Name: Supplementary Data 7
Description: Single gene scores the KRAS synthetic lethal screens and media condition screens in isogenic cells.

File Name: Supplementary Data 8
Description: E-MAP scores for gene pairs in KRAS G12D cells.

File Name: Supplementary Data 9
Description: Scores for drug screen in KRAS mutant versus control eGFP cell lines.
