# Supplementary figures and images for: Integration of multiple biological contexts reveals principles of synthetic lethality that affect reproducibility
Source: Nat Commun. 2020 May 12;11:2375. doi: 10.1038/s41467-020-16078-y (PMC7217969; doi:10.1038/s41467-020-16078-y)

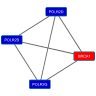

Supplement: Supplementary file 6 — Supplementary Data 3 [file 41467_2020_16078_MOESM6_ESM.zip › CytoscapeSession-2019_09_28-13_36/session_thumbnail.png]

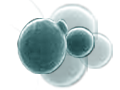

Supplement: Supplementary file 6 — Supplementary Data 3 [file 41467_2020_16078_MOESM6_ESM.zip › CytoscapeSession-2019_09_28-13_36/apps/org.cytoscape.ding.customgraphicsmgr/1.png]

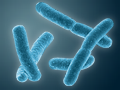

Supplement: Supplementary file 6 — Supplementary Data 3 [file 41467_2020_16078_MOESM6_ESM.zip › CytoscapeSession-2019_09_28-13_36/apps/org.cytoscape.ding.customgraphicsmgr/10.png]

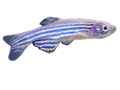

Supplement: Supplementary file 6 — Supplementary Data 3 [file 41467_2020_16078_MOESM6_ESM.zip › CytoscapeSession-2019_09_28-13_36/apps/org.cytoscape.ding.customgraphicsmgr/11.png]

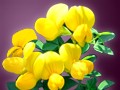

Supplement: Supplementary file 6 — Supplementary Data 3 [file 41467_2020_16078_MOESM6_ESM.zip › CytoscapeSession-2019_09_28-13_36/apps/org.cytoscape.ding.customgraphicsmgr/12.png]

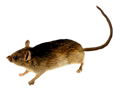

Supplement: Supplementary file 6 — Supplementary Data 3 [file 41467_2020_16078_MOESM6_ESM.zip › CytoscapeSession-2019_09_28-13_36/apps/org.cytoscape.ding.customgraphicsmgr/13.png]
