# Supplementary material for: Integration of multiple biological contexts reveals principles of synthetic lethality that affect reproducibility
Source: Nat Commun. 2020 May 12;11:2375. doi: 10.1038/s41467-020-16078-y (PMC7217969; doi:10.1038/s41467-020-16078-y)
Supplement: Supplementary file 13 — Reporting Summary [file 41467_2020_16078_MOESM13_ESM.pdf]

nature research

Corresponding author(s): Bandyopadhyay

Last updated by author(s): Mar 12, 2020

Reporting Summary

Nature Research wishes to improve the reproducibility of the work that we publish. This form provides structure for consistency and transparency in reporting. For further information on Nature Research policies, see Authors & Referees and the Editorial Policy Checklist .

Statistics

For all statistical analyses, confirm that the following items are present in the figure legend, table legend, main text, or Methods section.

n/a	Confirmed	
☐	☒	The exact sample size (*n*) for each experimental group/condition, given as a discrete number and unit of measurement
☐	☒	A statement on whether measurements were taken from distinct samples or whether the same sample was measured repeatedly
☐	☒	The statistical test(s) used AND whether they are one- or two-sided<br>*Only common tests should be described solely by name; describe more complex techniques in the Methods section.*
☐	☒	A description of all covariates tested
☐	☒	A description of any assumptions or corrections, such as tests of normality and adjustment for multiple comparisons
☐	☒	A full description of the statistical parameters including central tendency (e.g. means) or other basic estimates (e.g. regression coefficient) AND variation (e.g. standard deviation) or associated estimates of uncertainty (e.g. confidence intervals)
☐	☒	For null hypothesis testing, the test statistic (e.g. *F*, *t*, *r*) with confidence intervals, effect sizes, degrees of freedom and *P* value noted<br>*Give P values as exact values whenever suitable.*
☒	☐	For Bayesian analysis, information on the choice of priors and Markov chain Monte Carlo settings
☒	☐	For hierarchical and complex designs, identification of the appropriate level for tests and full reporting of outcomes
☒	☐	Estimates of effect sizes (e.g. Cohen's *d*, Pearson's *r*), indicating how they were calculated

Our web collection on statistics for biologists contains articles on many of the points above.

Software and code

Policy information about availability of computer code

Data collection	Cell count information was collected using Thermo Cellomics Scan software (Version 6.4.3).
Data analysis	Statistical analyses for this study were performed using GraphPad Prism 6 (version 6.0g), R (version 3.32) and gProfiler (https://biit.cs.ut.ee/gprofiler/). Images were analyzed using imageJ (https://imagej.nih.gov/ij/).

For manuscripts utilizing custom algorithms or software that are central to the research but not yet described in published literature, software must be made available to editors/reviewers. We strongly encourage code deposition in a community repository (e.g. GitHub). See the Nature Research guidelines for submitting code & software for further information.

Data

Policy information about availability of data

All manuscripts must include a data availability statement. This statement should provide the following information, where applicable:

- Accession codes, unique identifiers, or web links for publicly available datasets
- A list of figures that have associated raw data
- A description of any restrictions on data availability

All network analysis raw and processed data are available as an excel sheet as well as a Cytoscape session file. CORUM was downloaded from (v3.0, https://mips.helmholtz-muenchen.de/corum/). HumanNet was from (http://www.functionalnet.org/humannet/) and iRefWeb from (http://wodaklab.org/iRefWeb/). For each published KRAS SL study, data was downloaded from the supplementary material from the respective study.

Field-specific reporting

Please select the one below that is the best fit for your research. If you are not sure, read the appropriate sections before making your selection.

Life sciences study design

All studies must disclose on these points even when the disclosure is negative.

Sample size	A minimum of four technical replicates was used for cell line analysis. Sample size was chosen accordingly to previous publications.
Data exclusions	N/A
Replication	Western blots were reproduced at least twice. All attempts to replicate were successful.
Randomization	N/A
Blinding	N/A

Reporting for specific materials, systems and methods

We require information from authors about some types of materials, experimental systems and methods used in many studies. Here, indicate whether each material, system or method listed is relevant to your study. If you are not sure if a list item applies to your research, read the appropriate section before selecting a response.

Materials & experimental systems

n/a	Involved in the study	
☐	☒	Antibodies
☐	☒	Eukaryotic cell lines
☒	☐	Palaeontology
☒	☐	Animals and other organisms
☒	☐	Human research participants
☒	☐	Clinical data

Methods

n/a	Involved in the study	
☒	☐	ChIP-seq
☒	☐	Flow cytometry
☒	☐	MRI-based neuroimaging

Antibodies

Antibodies used	BRCA1(C-20) (Santa-Cruz, sc-672), total KRAS antibody (Sigma, 3B10-252) at 1:500, ERK1/2 (p44/42) antibody (CST, #9102), Phospho-ERK1/2 (phospho-p44/p42) (CST, #9101), beta-Actin (CST, #3700) at 1:2500 and Anti-Histone γ-H2AX (Sigma,#05-636), Goat anti-Mouse Alexa Fluor 647 Polyclonal (Invitrogen, #A-21236) at 1:1000.
Validation	Antibodies were described and validated in this and previous publications referenced in this manuscript.

Eukaryotic cell lines

Policy information about cell lines

Cell line source(s)	MCF10A cells were purchased from ATCC. C5N and CCH85 cells were generated and provided by the Balmain Laboratory.
Authentication	MCF10A cells were authenticated using STR.
Mycoplasma contamination	All cell lines used tested negative for mycoplasma
Commonly misidentified lines (See ICLAC register)	No commonly misidentified cell lines used.
